# Development and validation of an effective and sensitive technique for nitrate determination in fruits and vegetables using HPLC/PDA

**DOI:** 10.1186/s13065-023-01008-y

**Published:** 2023-08-24

**Authors:** Rayhan Uddin, G. M. Rabiul Islam, Mohammad Zia Uddin, Mostak Uddin Thakur

**Affiliations:** 1 Department of Food Engineering and Tea Technology, Shahjalal University of Science and Technology, Sylhet, 3114 Bangladesh; 2 Delta Pharma Limited, Pakundia Plant, Kishoreganj, 2300 Bangladesh; 3 Department of Analytical Chemistry and Environmental Science, Training Institute for Chemical Industries, Narsingdi, 1611 Bangladesh

**Keywords:** Nitrate, Development, Validation, Photo diode array, Fruits and vegetables, HPLC

## Abstract

This study aims to develop an effective and sensitive HPLC (High Performance Liquid Chromatography) method to determine the nitrate concentration in fruits and vegetables (F & V) using a C_18_ column (ZORBAX Eclipse XDB-C_18_, 80Å, 250 × 4.6 mm, 5 μm (Agilent Technologies)) maintained at 40 ^0^ C, a mobile phase made up of methanol and buffer (pentane sulfonic acid sodium salt solution), and a Photo Diode Array Detector (PDA) at 225 nm. The developed method is validated in terms of selectivity, linearity, accuracy, precision, suitability, the limit of detection (LOD), and the limit of quantification (LOQ) according to the European Union Decision 2002/657/EC. The result revealed that a ratio of 30: 70 of the organic modifier methanol and buffer with pH 2.8 shows the highest efficiency. The calibration curve shows linearity with a correlation coefficient (r) of 0.9985. The LOD and LOQ were found to be 2.26 mg/kg and 7.46 mg/kg. The recovery was in the range of 98.96–100.21%. Moreover, the greenness assessment scores of different approaches (eco-scale score of 76, AGREE score of 0.71, and few red shades in GAPI portray) were at a very excellent level. Thus, our developed method is fully validated and can determine the nitrate content in F & V.

**Supplementary Information:**

The online version contains supplementary material available at 10.1186/s13065-023-01008-y.

## Introduction

Nitrate is a typical compound found in the environment, which constitutes a significant part of the nitrogen cycle. It enters the human food chain through the soil, water, chemical fertilizer, and food additives [1]. Nitrate profoundly exists in fruits and vegetables and contributes to about 85% of the dietary intake of nitrate [2]. The occurrence of nitrate in the human food chain has long been acknowledged as a severe issue concerning the risk of methemoglobinemia and the formation of carcinogenic N-nitroso compounds [3–7].

However, in recent years, the importance of monitoring residual nitrate in the human diet, including fruits and vegetables, has changed. Some researchers are also highlighting the favorable effects of dietary intake of nitrate due to the discovery of the profound significance of nitric oxide, a derivative of nitrate, in many physiological systems. The dietary consumption of nitrate exhibits many health benefits, including reduced risk of cardiovascular disease, reduction of blood pressure, stroke, renal failure, gastric ulcer, myocardial infarction, and metabolic syndrome [8–10]. No epidemiological evidence is present currently to link nitrate with cancer [11, 12]. It has also been hypothesized that the antioxidants and other nutritional content found in fruits and vegetables make them less likely to have adverse effects and more likely to provide beneficial ones [13, 14].

Therefore, the effect of nitrate emerges as a bargaining issue, as is the case for so many food items and nutrients. Sometimes, nitrate can be harmful at high levels, while it can be beneficial at other levels. An ADI for dietary nitrate was established in 2002 by the Joint FAO/WHO Expert Committee on Food Additives (JECFA) at a level of 3.7 mg NO_3_^–^ per kg of body weight to avoid appreciable health risks [15]. Hence the monitoring of nitrate in fruits and vegetables is apparent.

Various analytical methods have been employed to identify and quantify nitrate in fruits and vegetables, including HPLC, spectrophotometry, ion exclusion chromatography, capillary electrophoresis, liquid chromatography, and ion chromatography [16–21]. Nevertheless, many of the methods described in specialized literature are very trying, less sensitive, require many reagents, are time-consuming, and have drawbacks. In recent years, the ion chromatography (IC) approach with conductivity or UV detection has been the most widely used technique due to its speed, simplicity, and ruggedness [18, 22–24] and appeared as a better alternative to HPLC techniques in anion including NO_3_^−^, NO_2_^−^, Cl^−^ etc. and different drugs & pesticides analysis [25–28].

However, some authors documented process selectivity issues, such as interfering compounds like sugar phosphate and chloride ion that have the same chromatographic behavior as the nitrate ion [29, 30]. For nitrate analysis, recent literature has reported using UPLC/MS due to their higher sensitivity [31]. However, this more sophisticated instrumentation cannot be employed for all analytical methods in routine laboratories where many analyses are usually required. Apart from this, due to its high price, lack of trained operating personnel, and complexities in the operating system make, its accessibility is very limited in different research laboratories, especially those in developing countries [32, 33]. Further, the fluorometric HPLC method requires a complicated and time-consuming sample preparation procedure to remove matrix components [34]. In addition, in this method, the long sample preparation time can introduce nitrite contamination from the environment or convert nitrate to nitrous acid due to its unstable nature [34].

UV/Vis detector is the most adaptable detector known for its wide linear range and simplicity [35, 36]. But these detectors face several flaws; for example, during the detection of nitrate, the absorbance is observed at 210 nm, which is particularly susceptible to interference from chloride that usually presents itself in fruits and vegetables [34, 37]. Though electrochemical detection is more robust than UV/Vis detection, it is subjected to chloride interference and is not practical for routine analysis due to its poor sensitivity [37]. However, a highly sensitive, selective, speedy, and accurate method for determining nitrate with minimal sample manipulation, analysis time and regents, and no interference in the detection spectra is required.

Diode-Array Detection (DAD) or Photodiode-Array Detection (PDA) is an analytical technique where the array of diodes can measure the entire wavelength spectrum in real-time [38]. Therefore, compared with the HPLC/UV/Vis detection, which generally only measures a couple of user-selectable specific wavelengths, HPLC/PDA may help distinguish analytes with different spectra. Hence, we developed and validated a rapid and sensitive HPLC method with PDA detection to determine nitrate.

## Materials and methods

### Experimental design

In the present study, preliminary trials were performed with the standard to optimize suitable chromatographic conditions for the determination of nitrate, which is well known as Response Surface Methodology (RSM) [39]. The trial was carried out with the organic mobile phase modifier, which plays a crucial role in separating nitrate with a symmetric peak and in determining the sensitivity of the chromatographic analytical method. In the organic modifier, the methanol and buffer ratios were chosen as 25:75, 30:70, and 35:65, respectively. The pH levels of the mobile phase were selected as 2.6, 2.8, and 3.0, respectively, and they both contributed as the independent parameters of the technique (Supplementary Table 1).

In the RSM, the dependent parameters were the peak area, theoretical plates, and tailing factor, which reduced study time and optimized the peak resolution of the system. To select the best peaks of HPLC, the peak area and the theoretical plates should be maximum, and the tailing factor should be as low as possible [39].

### Chemicals and reagents

We used analytical or HPLC-grade chemicals and reagents. HPLC grade methanol, HPLC grade 1-Pentanesulfonic acid sodium salt, analytical grade potassium nitrate (Merck, Germany), and analytical grade hydrochloric acid (Sigma Aldrich) were collected from an online supplier. Deionized water was used during the experiment as the solvent.

The organic mobile phase buffer solution was prepared by dissolving 1-Pentanesulfonic acid sodium salt (1.74 g) in 950 mL deionized water in a 1000 mL beaker. Further, deionized water was added to the mark. The standard KNO_3_ (162.70 mg) was weighed in a 100 mL volumetric flask and then dissolved in deionized water to prepare a 1000 ppm nitrate solution. The working standard solutions were designed by further diluting the standard stock solution.

### Instrumentation and chromatographic condition

Analysis was performed using HPLC (Shimadzu HPLC Prominence-*i* LC-2030 LT) with Photo Diode Array (PDA) detector commanded by lab solution software. The separation was carried out on a C_18_ column (ZORBAX Eclipse XDB-C_18_, 80Å, 250 × 4.6 mm, 5 μm (Agilent Technologies). The standard and sample injection volume was 10 μL with a flow rate of 1 mL/min and a wavelength of 225 nm (Chosen through the peak purity testing from a wavelength range of 200–260 nm). The column oven temperature was 40 ^0^ C, and the run time was 10 min. The analysis was performed isocratically.

### Sample preparation and extraction

**Fig. 1:**
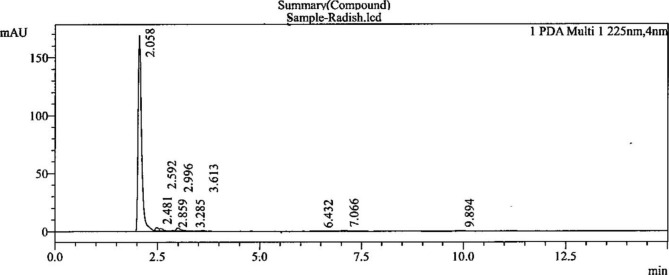
Chromatogram of nitrate in radish sample

We used radish as a sample to develop the method in this study. The non-edible parts of the radish sample were removed and then subjected to cutting and homogenization. Then, the homogenized content was immediately stored at − 20 °C before the analysis. Of the homogenized content, 2 g was added to 50 mL of deionized water in a 100 mL volumetric flask to extract nitrate. The flask was then placed in a boiling water bath for 20 min at 80 °C, mixed by shaking, and kept on the table to cool down before further dilution to a final volume of 100 mL. Finally, 10 mL of the sample extract was passed through a 0.45 μ membrane filter. The first 3 mL of filtrate was discarded, and the rest was stored. The sample was analyzed within 1 h of preparation and extraction. Figure 1 shows the chromatograms of the nitrate in the radish sample. Using a standard calibration curve (Supplementary Fig. 1), the radish nitrate content was determined to be 2501 mg/kg.

### Method validation procedure

The method was validated according to the performance characteristics of European Commission Decision 2002/657/EC [40] and with the EU regulation 2017/625 [41]. The parameters such as linearity, system suitability, accuracy, precision (repeatability and reproducibility), the limit of detection (LOD), and the limit of quantification (LOQ) are evaluated [42].

Five different blank solutions (Eluent) were analyzed around the retention time of 2.018 min at wavelength 225 nm to verify the absence of other interfering compounds. The calibration curves were constructed using five different concentrations of nitrate (80, 90,100,110, 120 ppm). The linearity was calculated using the least-squares method to analyze a regression line representing the peak area as a function of the standard concentration. The linear calibration curve of the developed method can be expressed by the regression equation y = 17832.8*x + 93725.8 with the regression coefficient of 0.9999489 (see supplementary Fig. 1). The system suitability was determined by injecting 100 ppm standard solutions six times. Accuracy is defined as “the degree to which the result of a measurement conforms to the correct value or a standard” and refers to how close a measurement is to its agreed value. The accuracy study was performed at three concentration levels of 80 ppm, 100 ppm, and 120 ppm sample solution, i.e., the lower, middle, and higher concentrations. Three injections were performed for each concentration level, followed by calculating the retrieval/recovery efficiency. For preparing 100 ppm sample solution, 4 g of the radish sample encompassing 10 mg nitrate (calculated from the obtained nitrate during sample extraction) was added into 100 mL water. Similarly, for 80 ppm and 120 ppm concentration levels, 3.2 and 4.8 g of radish are added in 100 mL water, respectively.

We determined the repeatability using six injections of the sample solution having the same concentration of 0.1 mg/mL nitrate to verify the **precision** of the nitrate assay method. The **intermediate precision-1** was determined in terms of reproducibility by injecting six sample solutions with the same concentration of 0.1 mg/mL nitrate after seven days of method precision study using the same sample and the analytical instruments used for method precision study. The **intermediate precision-2** reveals the degree of reproducibility of test results obtained by analyzing the same samples under varying conditions (viz., slight change of flow rate and operating temperature) and is determined using six injections of the sample solution with the same concentration of 0.1 mg/mL nitrate by the same analyst.

We also performed the **ruggedness** test, which is the measurement of reproducibility performed in the same environmental and operational condition by another analyst since analyst-to-analyst results may vary. The results for this test are obtained by injecting the same solution six times (0.1 mg/mL).

In this study, the LOD and LOQ were also performed to understand the method sensitivity. LODs were calculated as the lowest observable concentration resulting in a signal-to-noise ratio of 3:1. The LOQs were estimated as the concentration giving a signal-to-noise ratio of 10:1. We calculated both the LOD and LOQ using repeated injections (n = 6) of the identical concentration (100 ppm) of standard solution.

Furthermore, we considered the greenness analytical chemistry concept and assessed the greenness of the method. This concept refers to the development of any eco-friendly technique which encompasses approaches like reducing hazardous substances, decreasing waste, increasing occupational safety, and reducing energy consumption [43]. All the mentioned approaches are usually consulted to make any analytical method inclined towards the fulfillment of sustainability aspects.

However, several tools are available to assess the greenness of any method, for example, Green Analytical Procedure Index (GAPI), National Environmental Method Index (NEMI), Analytical Eco-scale, Analytical Method Greenness Score (AMGS), and Analytical greenness (AGREE) metric [44–47]. Here, we employed the widely accepted and comprehensive analytical Eco-scale score method, GAPI and AGREE tools to evaluate the greenness of the proposed method of the present study [48–50]. The approach in Eco scale score involves assigning penalty points to different parameters considering their negative impact on the environment, followed by subtracting the total penalty score from 100 to obtain the Eco scale score [49, 50]. It is known/considered that when the eco scale value is closer to 100 it is considered greener, and hence criteria are set for the method in greenness analysis as ‘Ideal’ if the Eco scale score = 100, ‘Excellent’ if the score is > 50 and ‘Inadequate’ if the score is < 50. The GAPI tool facilitates qualitative assessment of greenness of any analytical method. It encompasses consideration of 15 parameters followed by representation of a five-pentagons symbol [47, 51]. The GAPI pictogram contains 3 color i.e. green, yellow and red which symbolize respectively low, medium and high impact of the respective parameters on the environment [44, 50]. In this method the greenness level usually visually appear through the color combination of the pictogram. If fewer non-green elements (i.e., reddish) and the more greenest and least green element (i.e., green and yellow tints) appear then it illustrate the excellent level of eco-friendliness of any analytical method [51].The other approach was the AGREE metric which covers all the 12 principles of green analytical chemistry [52]. It encompasses assignment of weightage to all principles to transform greenness into a unified scale of 0–1 and finally presenting the total greenness score in center after considering assessment result of each principle. The greenness of any method relies on the closeness of the score to 1 [47, 51].

### Statistical analysis

We validated the method by calculating the relative standard deviation (RSD). In RSM, to optimize the best experimental conditions, we used a 3-level 2-factor face-centered central composite design (3^2^ CCD) [53]. Variance analysis was employed in the quadratic polynomial model to determine the peak area, theoretical plate, and tailing factors. The determination coefficient (r^2^ statistic) was observed to evaluate the accuracy and general ability of the quadratic polynomial model. All the analysis was carried out using Minitab 17 statistical software (Minitab Inc. PA. 2010).

## Results

### Development and optimization of the chromatographic condition

**Fig. 2:**
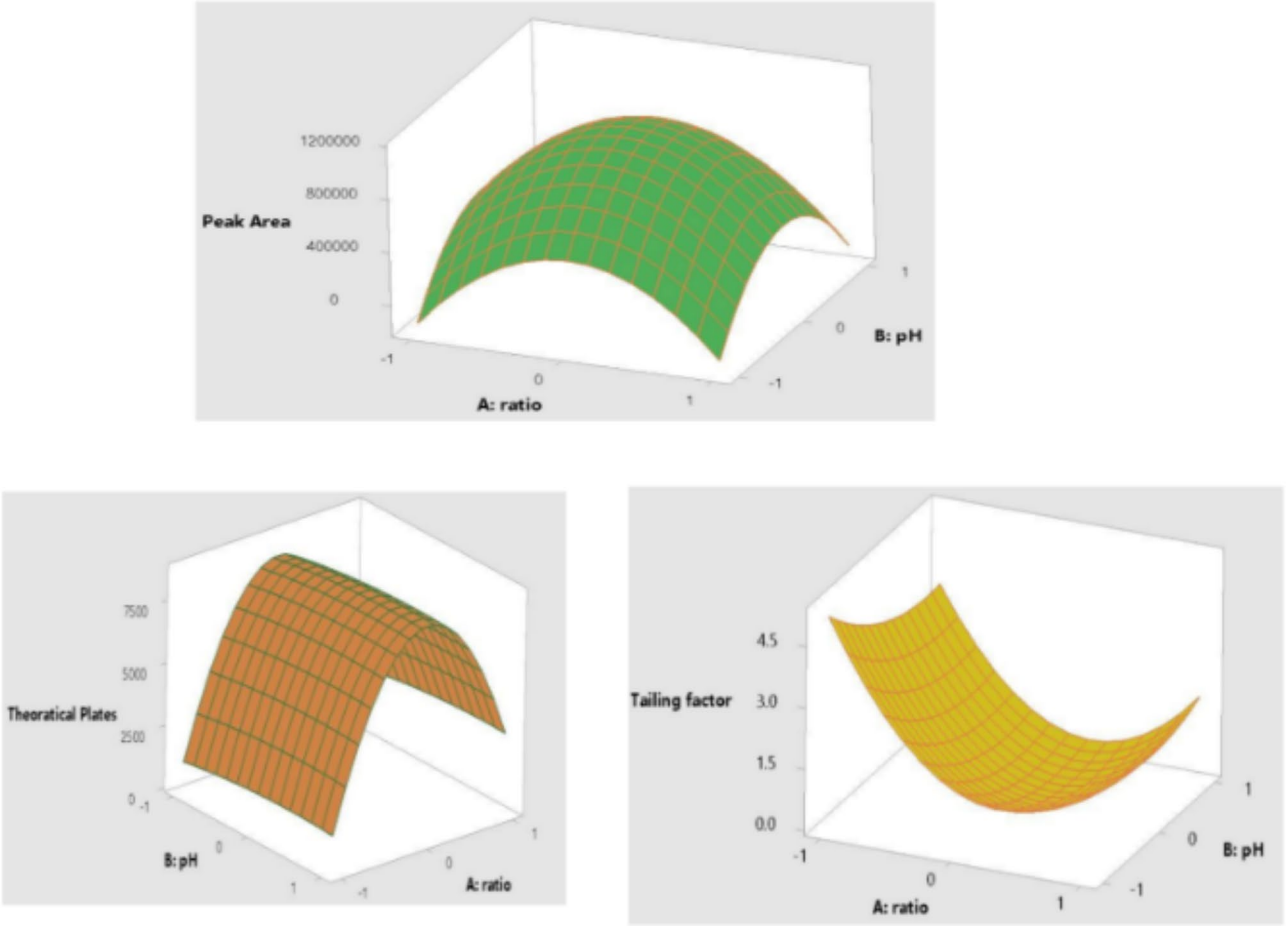
Three dimensional surface response presenting the effect of ratio and pH on peak area, theoretical plates and tailing factor

We tried to optimize the concentration of the organic modifier and pH at various ratios. Considering the peak area, theoretical plates, and tailing factor, the highest sensitivity was observed at a ratio of 30:70 and pH of 2.7 (Fig. 2) (Data shown in Supplementary Table 2). The determination coefficient obtained (r^2^ statistic) indicates that the model can explain 78.71%, 99.81%, and 95.15% of the variability of the data regarding peak area, theoretical plate, and tailing factors, respectively.

### Method validation results

**Fig. 3:**
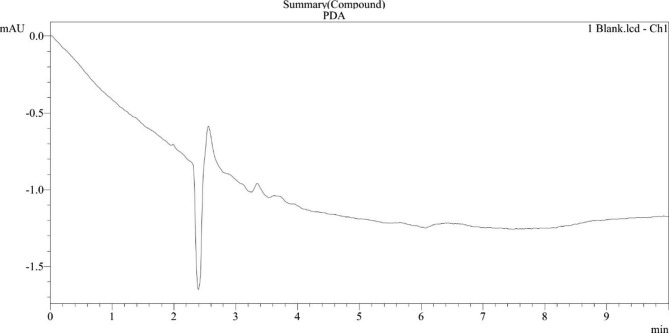
Peak area from the blank analysis (specificity analysis)

During specificity analysis, no endogenous compounds were found to interfere with the eluent. The blank analysis does not show any peak area to reflect any other interfering compounds (Fig. 3). The data acquisition for the system suitability is measured using the retention time and peak areas of six consecutive injections. The result shows that the RSD is less than 2% in both cases, thereby indicating the system’s good performance (Supplementary Table 3). The findings of the accuracy tests are presented in Table 1 (Results shown under Supplementary Table 4).

**Table 1:** Evaluation of the accuracy of the developed method

Concentration levels	No. of observations	Weight of nitrate (Equivalent sample) (mg/100 ml)	Resulted in quantity (mg)	Recovery of the sample (%)	Average (%)	SD (%)	RSD (%)
	1	8.00	8.030	**100.186**			
80 ppm	2	8.10	8.016	**98.958**			
	3	8.00	8.017	100.207			
	1	10.00	9.916	99.161			
100 ppm	2	9.90	9.908	100.081	99.6023	0.5165	0.5185
	3	10.00	9.918	99.179			
	1	12.00	11.973	99.779			
120 ppm	2	12.10	11.983	99.034			
	3	12.00	11.980	99.836			

The findings exhibit that the recovery ranged from 98.96 to 100.19%, with an RSD of 0.52%. The outputs from the precision and intermediate precision-1 tests revealed RSD of 0.38% and 0.43%, respectively (see Supplementary Tables 5 & 6). The intermediate precision-2 scored RSD < 2% with an average of 99.45% recovery (Table 2).

**Table 2:** Evaluation of intermediate precision-2 of the developed method

No. of observations	Weight of the sample (nitrate equivalent ) mg/100 ml	Resulted in quantity (mg/100 ml)	Recovery of the sample (%)	Average (%)	SD (%)	RSD (%)
1	10.00	9.939	99.391			
2	10.10	9.998	98.992			
3	10.00	9.977	99.766	**99.4562**	0.3227	0.3245
4	10.00	9.979	99.793			
5	10.00	9.960	99.603			
6	10.10	10.018	99.192			

The outcomes obtained from the determination of the three precisions reveal that they are reproducible. The ruggedness test shows that the nitrate estimations do not notably affect the change of analysts as the values of % RSD were within the allowed limits of 2% (Table 3). We found the LOD and LOQ to be 2.26 mg/kg, and 7.46 mg/kg, respectively.

**Table 3:** Evaluation of ruggedness of the developed method (analysis by another person)

No. of observations	Amount (mg/100 ml)		Recovery of the sample (%)	Average (%)	SD (%)	RSD (%)
	Sample taken	Resulted in quantity				
1	10.00	10.013	100.126			
2	10.10	10.070	99.705			
3	10.10	10.020	99.208	99.600	0.4049	**0.4065**
4	10.10	10.012	99.131			
5	10.10	10.097	99.971			
6	10.10	10.097	99.456			

### Results of greenness assessment of the method

Detailed strategies to assess the greenness of the method using analytical Eco scale are presented in Table 4. The penalty points of all potential steps of the methodology are listed and calculated. As can be seen in the table, the total calculated penalty point is 24, and so finally, the analytical eco-scale score is 76.

**Table 4:** Penalty points allotment and analytical eco-scale score calculation to assess the greenness of the present method

Reagents/Instrumentation	Amount	Amount’s penalty point	Hazard pictogram	Penalty points
**Reagents**				
KNO_3_	162.10 mg < 10 g	1	1	2
HCl	10–100 ml	2	2	4
Water	> 100 ml	3	0	0
Methanol	10–100 ml	2	3	6
Penta sulfonic acid sodium salt	1.74g < 10g	1	1	1
**Instrumentation**				
HPLC-PDA (Hermetically sealed device & proper lab practice adopted by analyst)				0
Sample preparation (storage and boiling water bath)				3
**Occupational hazard**				0
**Waste**				
Unused sample extract & non-edible part of the radish	> 10g			5
No treatment				3
**Total Penalty points**				24
**Analytical eco-scale score**				**76**

The associated parameters and final output in greenness assessment using GAPI tools and AGREE software are presented in Table 5. It can be observed that the AGREE score is 0.71 and in portrayed GAPI pictogram 6 fields are shaded green, 7 are in yellow and 2 are in red color.

**Table 5:** Greenness assessment using GAPI and AGREE tools

GAPI	AGREE
Categories with adopted strategies	Aspects with adopted strategies
**Sample preparation** 1. Collection: On line 2. Preservation: None 3. Transport: None 4. Storage: Under special conditions 5. Type of the method (direct/indirect): Simple procedure 6. Scale of extraction: Nano-extraction 7. Solvents/Reagents: Green solvents/reagents (only water) 8. Additional treatments: None **Reagents and solvents** 9. Amounts: 1-100 mL (1- 100 g) 10. Health hazard: HCl can cause serious or permanent injury, National Fire Protection Association (NFPA) health hazard rating = 3 11. Safety hazard: Flammability score of methanol = 3 and HCl = 0 **Instrumentation** 12. Energy: ≤0.1 kWh/sample 13. Occupational hazard: Hermetic sealing of the analytical process 14. Waste: Waste generation by HPLC methods is 1–10 ml (mainly, unused extract & non-edible part of the radish was the waste) 15. Waste treatment: No treatment	1. Select the sampling procedure: On line 2. Minimal sample size: >100 mg 3. In Situ: On line 4. Integration of analytical process and operational energy: < 3 steps 5. Miniaturized method: Semi-automated, as sample needed to be prepared 6. Derivatization: No, since microextraction wasn’t involved 7. Analytical waste: Reagents waste can be considered as less 8. The multi-analyte or multi parameters method: Single 9. The use of energy should be minimized: less 10. Reagents type: Some are biobased 11. Toxic reagents: Moderate level toxicity of HCl & methanol 12. Operator safety: No issue; Every potential risk has been avoided
GAPI pictogram	AGREE metric

## Discussion

Response surface methodology (RSM) provides the required statistical tools for designing and analyzing experimental trials for process optimization [54]. On evaluating the effect of the independent parameters on the dependent parameters using 3^2^ CCD, we found that the highest sensitivity was obtained at a concentration of organic modifier methanol: buffer at 30:70 and a pH of 2.8.

Through a complete validation procedure, according to the European Directives, the developed method proved to be selective, sensitive, and accurate [41]. The RSD to measure the system suitability, precision, intermediary precision, and ruggedness analysis is supposed to be less than 2%. Our study shows that RSD is within the acceptable criteria in all the cases.

This study revealed that the estimated LOD and LOQ were 2.26 mg/kg and 7.46 mg/kg, respectively, which is lower than the observed LOD and LOQ by Pardo-Marín et al. [55] (LOD = 7 mg/kg, and LOQ = 20 mg/kg) and Chung et al. [56] (LOD = 4 mg/kg, and LOQ = 20 mg/kg). These results imply that our method encompasses better detection and quantification of nitrate. Moreover, the recovery of our study is in the range of 98.9 to 100.21% which is an excellent result in comparison with the recovery result of Pardo-Marín et al. [55] (90–120%), Chung et al. [56] (83–106%), and Merino [57] (70–110%). The developed HPLC method of de Kleijn and Hoven [58] revealed an excellent LOD of 103 ng/kg; however, it had a run time of more than 20 min making it costly with high mobile phase consumption. The Capillary Ion Chromatography (CIC) analytical method was introduced by D’Amore et al. [30], which showed recovery of 93–105% and LOQ of 3.65 mg/kg. The developed method of Siu and Henshall [59] took 10 min for the analysis with a recovery of around 90% and a LOD of 500 μg/kg; thus, it can be postulated that our method is better in the aspects of sample recovery (98.9 to 100.21%) and LOD (2.26 mg/kg). Few spectrophotometric methods also exist, such as Zatar et al. [60] and Alonso et al. [61], which take more than 30 min of analysis time. Here, in our present study, we observed a retention time of 2.028 min with excellent recovery. On the contrary, the UPLC–MS technique shows a speedy separation and a very low analysis time of 1 min with the LOD and LOQ values of 0.029 and 0.088 mg/kg [31]. This might be due to the highly sensitive and selective MS detector; however, this advanced instrumentation cannot be used for routine assays where a large number of measurements are required. Furthermore, from the greenness assessment of this method, we found the analytical eco scale score is 76, which is > 50, which confirms this technique as an excellent green methodology. This findings is in concord with few previous excellent HPLC green methodology for their respective intended purpose proposed by MoHaMed and Lamie [62] having score of 86, Delhiraj and Anbazhagan [45] having score of 60, Mohamed and Fouad [63] having score of 57, Peleshok et al. [64] having score of 84, and Emam and Abdelwahab [65] having score of 90. In addition, the AGREE score of 0.71 is also indicating quite an excellent level of greenness of the method with low environmental impact. In displayed GAPI pictogram the greater number of green & yellow and few red tinted shade combinations symbolizes that this method has less effect on the environment and thereby reveals the technique as an excellent greener approach. Both the AGREE score and GAPI pictogram are conforms with the AGREE score and portrayed GAPI pictogram from the study of Chen et al. [66] (score 0.74), Chaudhari et al. [67] (score 0.68), and Jagnade et al. [68] ( score 0.69), which are assessed & presented in review article of Kannaiah et al. [44]. However, the reasons for variation in our results compared to others are mostly due to the variability in chemical usage, instrumentation, operational safety, wastage, energy utilization, and other strategies associated with the techniques [44]. So, finally, considering the sustainability aspects, we can say that this method is worth being sustainable and an excellent alternative to analyze nitrate in F & V.

## Conclusion

This study presents a novel, sensitive, time-saving, and cost-effective HPLC method for analyzing nitrate content in fruits and vegetables. The method validation results, compliance with regulatory requirements, and high greenness score indicate its suitability for analysis and determination of nitrate in food products. Furthermore, the method’s precision, low solvent usage, and global availability of HPLC instruments in most research facilities emphasize its potential as an excellent alternative for routine assays for the intended purpose.

### Electronic supplementary material

Below is the link to the electronic supplementary material.

Supplementary Material 1

## Data Availability

The dataset analyzed during the current study is available from the corresponding author on reasonable request.
