# Supplementary material for: Development and validation of an effective and sensitive technique for nitrate determination in fruits and vegetables using HPLC/PDA
Source: BMC Chem. 2023 Aug 24;17(1):105. doi: 10.1186/s13065-023-01008-y (PMC10463336; doi:10.1186/s13065-023-01008-y)
Supplement: Supplementary file 1 — Supplementary Material 1 [file 13065_2023_1008_MOESM1_ESM.docx]

Supplementary Table 1. Experimental design for the developed method of HPLC

| Factors |  | Levels | | |
| --- | --- | --- | --- | --- |
| Independent | Symbol | -1 | 0 | 1 |
| Methanol: Buffer | A | 25:75 | 30:70 | 35:65 |
| pH of the mobile phase | B | 2.6 | 2.8 | 3.0 |
| Dependent |  |  |  |  |
| Peak Area |  |  |  |  |
| Theoretical plates |  |  |  |  |
| Tailing factor |  |  |  |  |

Supplementary Table 2. Experimental conditions and detected response values

| Exp  No | Run No | A | B | Peak area | Theoretical plates | Tailing factor |
| --- | --- | --- | --- | --- | --- | --- |
| 1 | 7 | -1 | -1 | 28775 | 1079.28 | 5.124 |
| 2 | 3 | 1 | -1 | 58871 | 2589.01 | 2.184 |
| 3 | 4 | -1 | 1 | 32558 | 1521.23 | 3.104 |
| 4 | 12 | 1 | 1 | 87752 | 3278.12 | 1.481 |
| 5 | 10 | -1 | 0 | 30255 | 1299.7 | 4.201 |
| 6 | 11 | 1 | 0 | 78998 | 2841.2 | 2.014 |
| 7 | 9 | 0 | -1 | 81587 | 8058.89 | 1.521 |
| 8 | 8 | 0 | 1 | 92982 | 8126.73 | 1.218 |
| 9 | 1 | 0 | 0 | 1208852 | 8587.9 | 0.184 |
| 10 | 2 | 0 | 0 | 1207452 | 8452.7 | 0.195 |
| 11 | 5 | 0 | 0 | 1207588 | 8512.7 | 0.225 |
| 12 | 6 | 0 | 0 | 1208794 | 8415.9 | 0.218 |
| 13 | 13 | 0 | 0 | 1208487 | 8542.1 | 0.207 |

Supplementary Table 3. Outcomes of the system suitability test

| Title | Sample Name | Sample ID | Ret. Time | Area |
| --- | --- | --- | --- | --- |
| System Suitability-1.lcd | NO_3_ | Standard | 2.032 | 1886820 |
| System Suitability-2.lcd | NO_3_ | Standard | 2.034 | 1894550 |
| System Suitability-3.lcd | NO_3_ | Standard | 2.025 | 1899287 |
| System Suitability-4.lcd | NO_3_ | Standard | 2.024 | 1899094 |
| System Suitability-5.lcd | NO_3_ | Standard | 2.025 | 1896268 |
| System Suitability-6.lcd | NO_3_ | Standard | 2.026 | 1896842 |
| Average |  |  | 2.028 | 1895477 |
| %RSD |  |  | 0.213 | 0.243 |

Supplementary Table 4. Data for determination of the accuracy

| No of observations | | Weight of the standard  (mg/100 ml) | Weight of nitrate (Equivalent sample)mg/100 ml) | Standard solution areas | Sample solutions areas | Resulted quantity  (mg) | Recovery of the sample (%) |
| --- | --- | --- | --- | --- | --- | --- | --- |
|  | 1 | 8.10 | 8.00 | 1535604 | 1532036 | 8.030 | 100.186 |
| 80 ppm | 2 | 8.10 | 8.10 | 1535604 | 1532163 | 8.016 | 98.958 |
|  | 3 | 8.10 | 8.00 | 1535604 | 1532353 | 8.017 | 100.207 |
|  | 1 | 10.00 | 10.00 | 1907167 | 1906799 | 9.916 | 99.161 |
| 100 ppm | 2 | 10.00 | 9.90 | 1907167 | 1905244 | 9.908 | 100.081 |
|  | 3 | 10.00 | 10.00 | 1907167 | 1907148 | 9.918 | 99.179 |
|  | 1 | 12.10 | 12.00 | 2243789 | 2238675 | 11.973 | 99.779 |
| 120 ppm | 2 | 12.10 | 12.10 | 2243789 | 2240490 | 11.983 | 99.034 |
|  | 3 | 12.10 | 12.00 | 2243789 | 2239961 | 11.980 | 99.836 |
|  | | | **Standard Potency** | | **99.18%** |  | |

% Recovery of sample = $\frac{Sample area \times Standard dilution \times Standard Potency \times100}{Standard area \times sample dilution}$

Resulted quantity = $\frac{Sample taken \times\% of recovery found}{100}$

**For 80 ppm sample solution**

| Recovery of Sample-1: | 1532036 | × |  | 8.10 |  | × |  |  | 100 |  | × |  | 99.18 | × | 100 | = | 100.186 | % |
| --- | --- | --- | --- | --- | --- | --- | --- | --- | --- | --- | --- | --- | --- | --- | --- | --- | --- | --- |
|  | 1535604 | × |  | 100 |  | × |  |  | 8.0 |  | × |  | 100 |  |  |  |  |  |

| Resulted Quantity = | 8.0 | × | 100.186 | = | 8.030 | mg |
| --- | --- | --- | --- | --- | --- | --- |
|  | 100 | | |  |  |  |

| Recovery of Sample-2: | 1532163 | × |  | 8.10 |  | × |  |  | 100 |  | × |  | 99.18 | × | 100 | = | 98.958 | % |
| --- | --- | --- | --- | --- | --- | --- | --- | --- | --- | --- | --- | --- | --- | --- | --- | --- | --- | --- |
|  | 1535604 | × |  | 100 |  | × |  |  | 8.10 |  | × |  | 100 |  |  |  |  |  |

| Resulted Quantity = | 8.10 | × | 99.958 | = | 8.016 | mg |
| --- | --- | --- | --- | --- | --- | --- |
|  | 100 | | |  |  |  |

| Recovery of Sample-3: | 1532353 | × |  | 8.10 |  | × |  |  | 100 |  | × |  | 99.18 | × | 100 | = | 100.207 | % |
| --- | --- | --- | --- | --- | --- | --- | --- | --- | --- | --- | --- | --- | --- | --- | --- | --- | --- | --- |
|  | 1535604 | × |  | 100 |  | × |  |  | 8.0 |  | × |  | 100 |  |  |  |  |  |

| Resulted Quantity = | 8.0 | × | 100.207 | = | 8.017 | mg |
| --- | --- | --- | --- | --- | --- | --- |
|  | 100 | | |  |  |  |

**For 100 ppm sample solution**

| Recovery of Sample-1: | 1906799 | × |  | 10 |  | × |  |  | 100 |  | × |  | 99.18 | × | 100 | = | 99.161 | % |
| --- | --- | --- | --- | --- | --- | --- | --- | --- | --- | --- | --- | --- | --- | --- | --- | --- | --- | --- |
|  | 1907167 | × |  | 100 |  | × |  |  | 10 |  | × |  | 100 |  |  |  |  |  |

| Resulted Quantity = | 10 | × | 99.161 | = | 9.916 | mg |
| --- | --- | --- | --- | --- | --- | --- |
|  | 100 | | |  |  |  |

| Recovery of Sample-2: | 1905244 | × |  | 10 |  | × |  |  | 100 |  | × |  | 99.18 | × | 100 | = | 100.081 | % |
| --- | --- | --- | --- | --- | --- | --- | --- | --- | --- | --- | --- | --- | --- | --- | --- | --- | --- | --- |
|  | 1907167 | × |  | 100 |  | × |  |  | 9.90 |  | × |  | 100 |  |  |  |  |  |

| Resulted Quantity = | 9.90 | × | 100.081 | = | 9.908 | mg |
| --- | --- | --- | --- | --- | --- | --- |
|  | 100 | | |  |  |  |

| Recovery of Sample-3: | 1907148 | × |  | 10 |  | × |  |  | 100 |  | × |  | 99.18 | × | 100 | = | 99.179 | % |
| --- | --- | --- | --- | --- | --- | --- | --- | --- | --- | --- | --- | --- | --- | --- | --- | --- | --- | --- |
|  | 1907167 | × |  | 100 |  | × |  |  | 10.00 |  | × |  | 100 |  |  |  |  |  |

| Resulted Quantity = | 10 | × | 99.179 | = | 9.918 | mg |
| --- | --- | --- | --- | --- | --- | --- |
|  | 100 | | |  |  |  |

**For 120 ppm sample solution**

| Recovery of Sample-1: | 2238675 | × |  | 12.10 |  | × |  |  | 100 |  | × |  | 99.18 | × | 100 | = | 99.779 | % |
| --- | --- | --- | --- | --- | --- | --- | --- | --- | --- | --- | --- | --- | --- | --- | --- | --- | --- | --- |
|  | 2243789 | × |  | 100 |  | × |  |  | 12.0 |  | × |  | 100 |  |  |  |  |  |

| Resulted Quantity = | 12.0 | × | 99.779 | = | 11.973 | mg |
| --- | --- | --- | --- | --- | --- | --- |
|  | 100 | | |  |  |  |

| Recovery of Sample-2: | 2240490 | × |  | 12.10 |  | × |  |  | 100 |  | × |  | 99.18 | × | 100 | = | 99.034 | % |
| --- | --- | --- | --- | --- | --- | --- | --- | --- | --- | --- | --- | --- | --- | --- | --- | --- | --- | --- |
|  | 2243789 | × |  | 100 |  | × |  |  | 12.10 |  | × |  | 100 |  |  |  |  |  |

| Resulted Quantity = | 12.10 | × | 99.034 | = | 11.983 | mg |
| --- | --- | --- | --- | --- | --- | --- |
|  | 100 | | |  |  |  |

| Recovery of Sample-3: | 2239961 | × |  | 12.10 |  | × |  |  | 100 |  | × |  | 99.18 | × | 100 | = | 99.836 | % |
| --- | --- | --- | --- | --- | --- | --- | --- | --- | --- | --- | --- | --- | --- | --- | --- | --- | --- | --- |
|  | 2243789 | × |  | 100 |  | × |  |  | 12.00 |  | × |  | 100 |  |  |  |  |  |

| Resulted Quantity = | 12.0 | × | 99.836 | = | 11.980 | mg |
| --- | --- | --- | --- | --- | --- | --- |
|  | 100 | | |  |  |  |

Supplementary Table 5. Data for precision

| No. of observations | Weight of the sample in mg | Sample Solutions Areas | Average area of the sample | Standard Deviation (SD) | RSD% |
| --- | --- | --- | --- | --- | --- |
| 1 | 10.00 | 1880395 |  |  |  |
| 2 | 10.00 | 1899077 |  |  |  |
| 3 | 10.00 | 1889364 | 1888254 | 7240.403 | 0.3834 |
| 4 | 10.00 | 1880308 |  |  |  |
| 5 | 10.00 | 1892572 |  |  |  |
| 6 | 10.00 | 1887807 |  |  |  |

Supplementary Table 6. Data for intermediate precision 1

| No. of observations | Weight of the sample in mg | Sample solutions areas | Average area of the sample | Standard Deviation (SD) | RSD% |
| --- | --- | --- | --- | --- | --- |
| 1 | 10.00 | 1881753 |  |  |  |
| 2 | 10.00 | 1892887 |  |  |  |
| 3 | 10.00 | 1901871 | 1888990.00 | 8038.4263 | 0.4255 |
| 4 | 10.00 | 1891078 |  |  |  |
| 5 | 10.00 | 1886144 |  |  |  |
| 6 | 10.00 | 1880207 |  |  |  |

Supplementary Fig. 1. Calibration curve for different concentrations of the standard
